# Supplementary material for: Rare regulatory mutations disrupt mesenchymal molecular programs driving endocardial cushion formation in bicuspid aortic valve
Source: Nat Commun. 2026 Apr 18;17:3587. doi: 10.1038/s41467-026-71758-5 (PMC13090386; doi:10.1038/s41467-026-71758-5)
Supplement: Supplementary file 1 — Supplementary Information [file 41467_2026_71758_MOESM1_ESM.pdf]

Rare regulatory mutations disrupt mesenchymal molecular programs driving endocardial cushion formation in bicuspid aortic valve

Supplementary Information

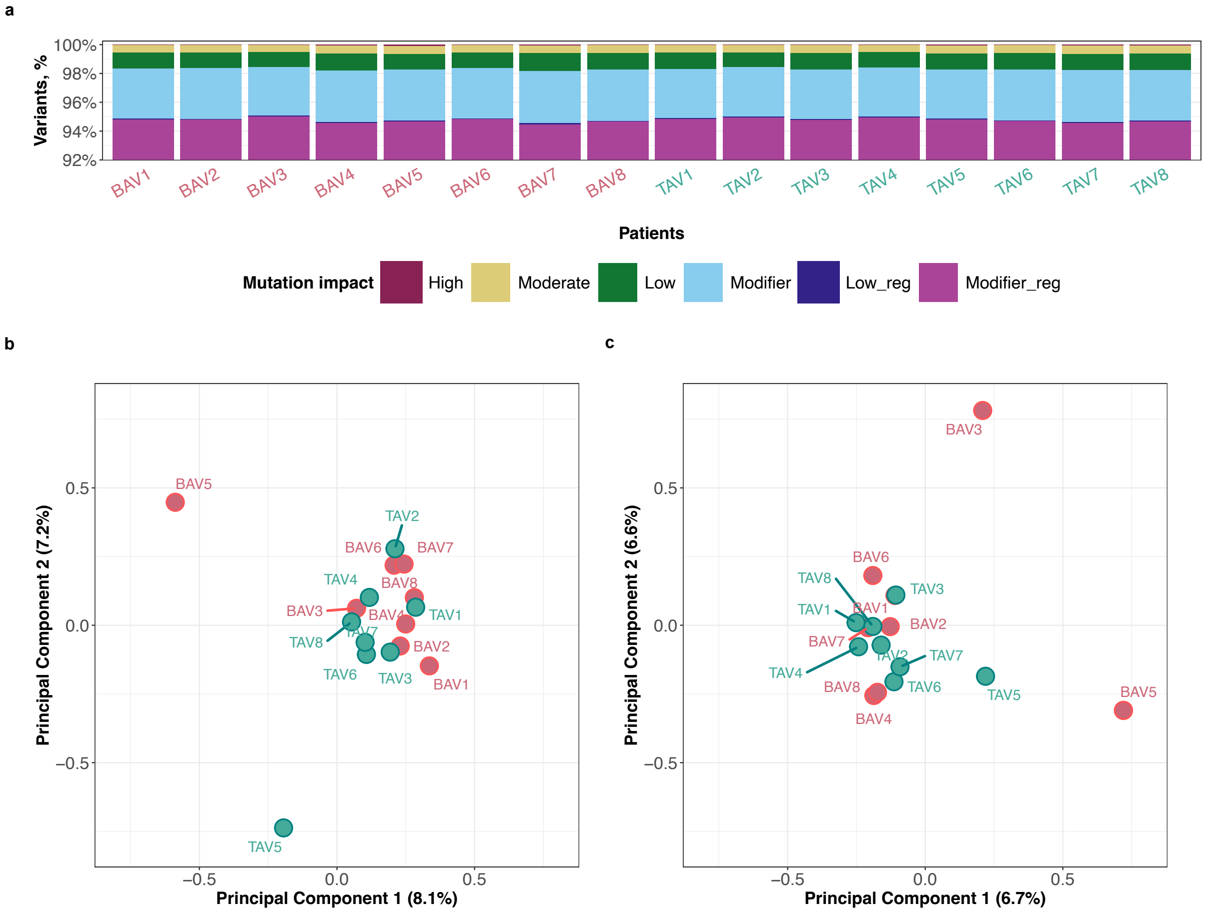

Supplementary Fig. 1: WGS analysis. a, Classification of short germline variants by impact and their coding or non-coding/regulatory status. **b,** PCA plot of high-impact protein-coding mutations, showing no distinct clustering between TAV and BAV groups. **c,** PCA plot of non-coding variants shows no distinct separation between TAV and BAV cohorts.

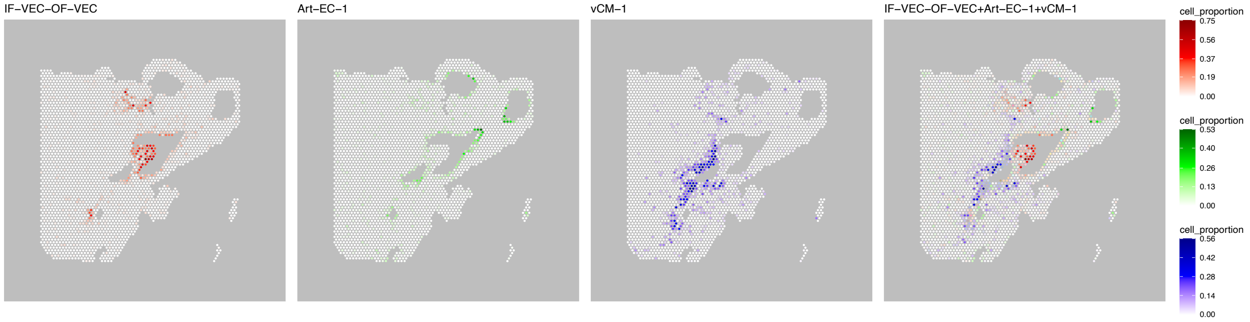

Supplementary Fig. 2: Non-mesenchymal cell states spatially relevant to the aortic valve region. Spatial mapping of cell states (proportion per Visium spot): IF_VEC + OF_VEC (red, combined), Art_EC_1 (green), vCM_1 (blue). Results are shown together to illustrate spatial relationships. Art_EC_1, endothelium of large arteries and veins; IF_VEC, inflow valve endothelial cell; OF_VEC, outflow valve endothelial cell; vCM_1, Ventricular cardiomyocytes.

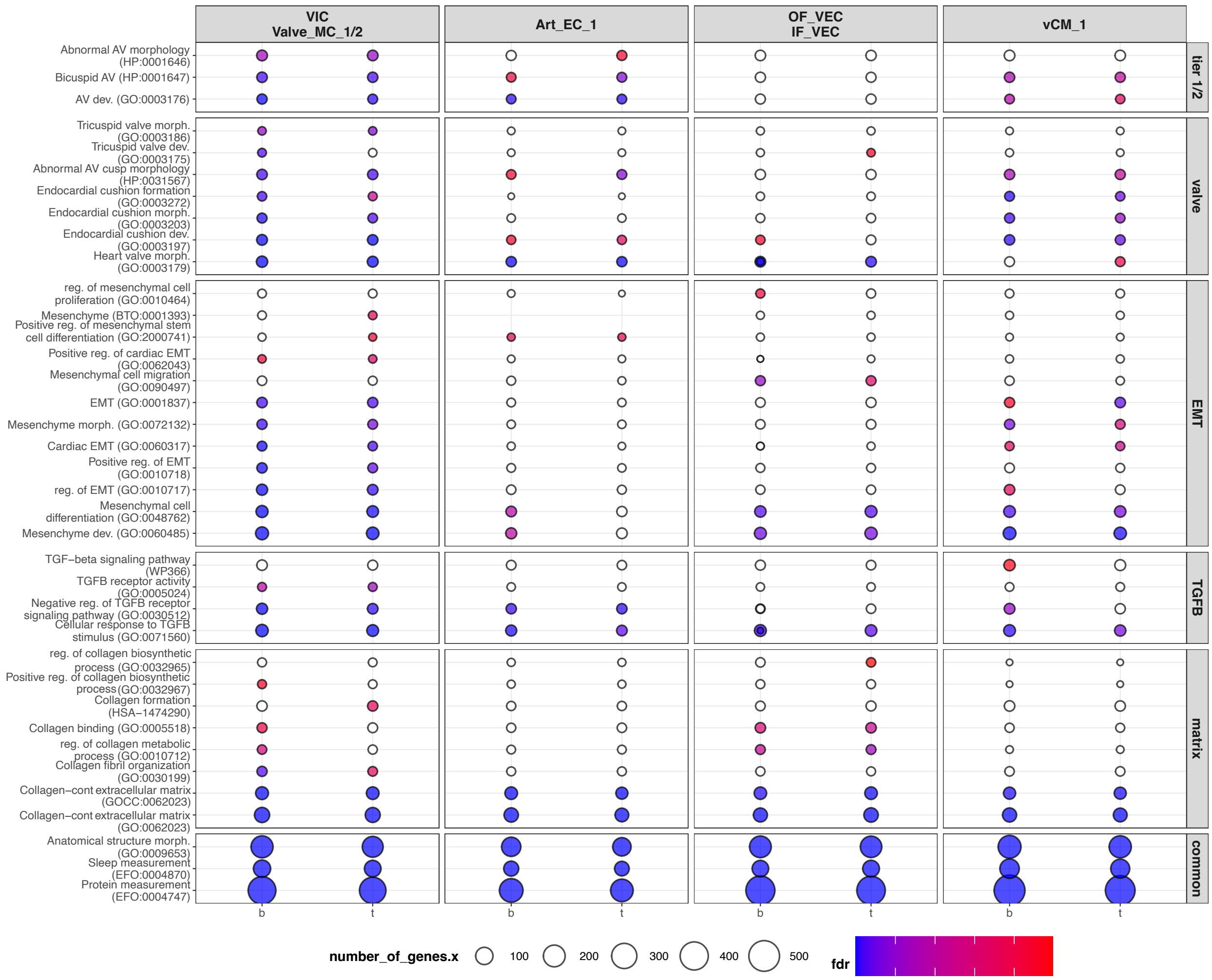

Supplementary Fig. 3: Gene set enrichment analysis of discordant cases. Gene set enrichment analysis of discordant genes across spatially relevant cell states. Art_EC_1, endothelium of large arteries and veins; IF_VEC, inflow valve endothelial cell; OF_VEC, outflow valve endothelial cell; VIC, valve interstitial cells; Valve_MC, cardiac valve-related mesenchymal cells; vCM_1, Ventricular cardiomyocytes.

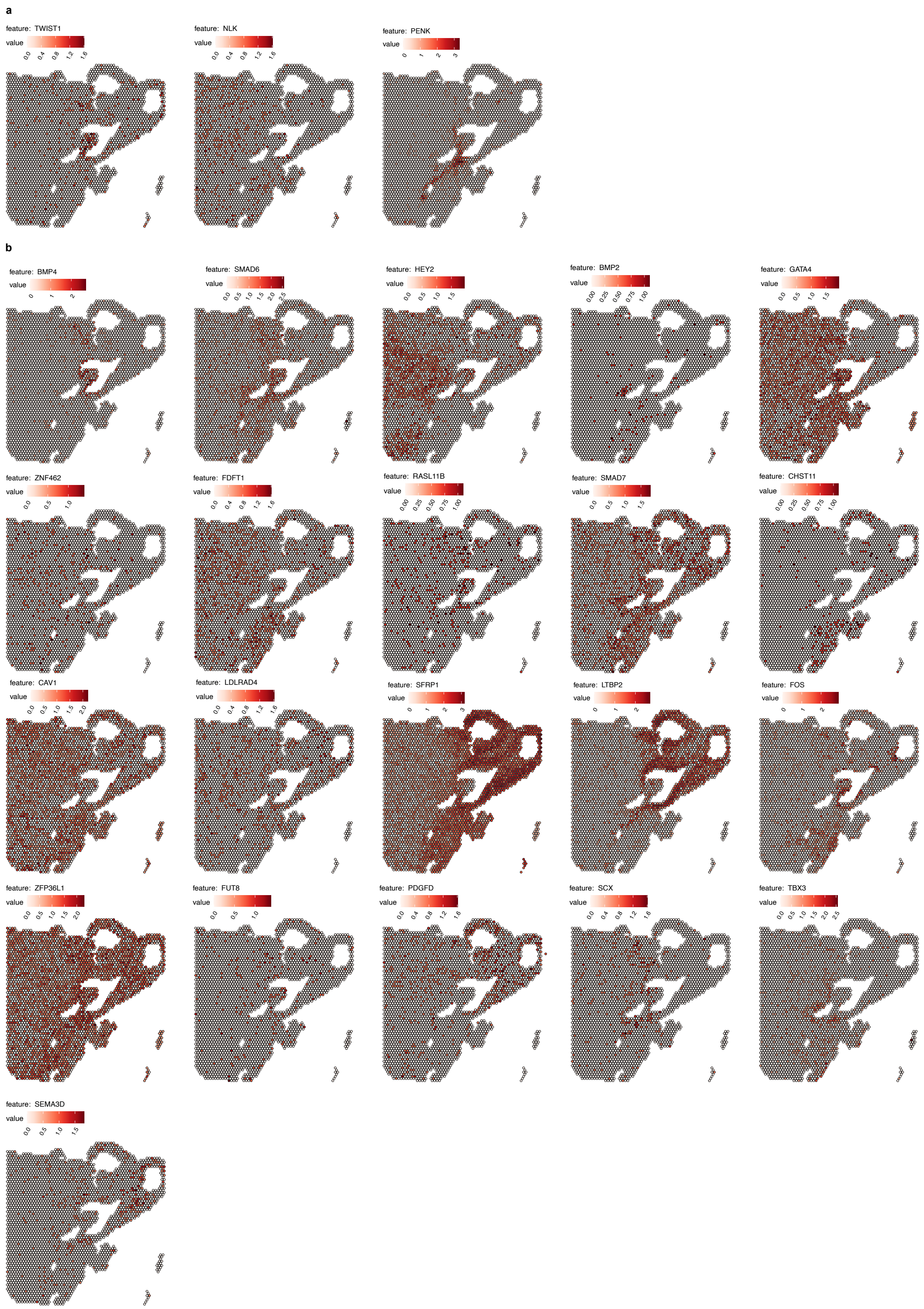

Supplementary Fig. 4: Spatial enrichment of tier 1 and 2, TGFβ and EMT gene. Genes from the TAV cohort (a) and BAV cohort (b) were visualized. BAV, bicuspid aortic valve; EMT, epithelial-to-mesenchymal transition; TAV, tricuspid aortic valve; TGFβ, transforming growth factor beta.

Supplementary Table 1. Patient Cohorts.

Patient ID	BAV fusion pattern	Monogenic Diseases
BAV1	Right-left	
BAV2	Right-left	
BAV3	Right-left	
BAV4	Equal cusps	
BAV5	NA	
BAV6	Right-left	
BAV7	Right-left	
BAV8	Right-left	
TAV1		
TAV2		
TAV3		Marfan syndrome
TAV4		
TAV5		
TAV6		
TAV7		
TAV8		

Supplementary Table 2. WGS sequencing statistics.

Samples	Total reads	Mapped reads	Duplicate reads	Mapped bases	Mean coverage	% genome covered ≥10×	% genome covered ≥20×	% genome covered ≥30×	Average quality
BAV1	649,758,962	648,072,806	60,181,310	96,870,496,610	27.3189	89.73	82.03	44.33	29.8
BAV2	997,110,633	994,692,439	94,101,302	148,705,052,064	41.8555	90.36	88.60	83.54	30.1
BAV3	673,452,835	671,732,011	65,111,305	100,416,382,662	28.1882	89.81	82.92	49.12	29.5
BAV4	1,250,284,149	1,248,064,446	47,486,397	125,114,512,698	37.4131	89.31	85.36	73.88	30.2
BAV5	437,585,036	436,324,456	13,931,234	43,433,818,199	13.0702	50.60	26.54	10.46	28.3
BAV6	1,218,751,861	1,215,810,853	49,065,865	121,637,964,398	36.2866	89.68	88.88	81.37	30.4
BAV7	791,052,475	789,496,794	28,775,842	78,632,532,289	23.5526	82.09	64.91	33.28	29.2
BAV8	1,687,789,868	1,683,770,150	62,801,727	168,334,795,550	50.3759	89.79	89.42	88.69	29.9
TAV1	680,750,763	678,416,797	56,053,304	101,043,191,703	28.816	89.50	86.46	52.58	30.4
TAV2	841,919,766	839,565,678	82,347,764	125,094,083,369	35.0755	89.61	88.79	78.75	31.0
TAV3	587,799,020	585,760,590	48,590,641	87,354,488,654	24.903	89.32	80.14	28.60	30.6
TAV4	781,329,857	779,699,779	30,128,431	77,889,344,535	23.2777	88.68	71.28	23.11	29.2
TAV5	581,411,273	580,422,102	19,663,746	57,833,657,758	17.3687	68.21	41.46	17.03	28.3
TAV6	1,011,556,741	1,009,587,572	35,065,729	101,044,691,189	30.3193	88.93	83.82	59.99	29.2
TAV7	886,128,684	884,665,272	32,726,827	88,625,468,040	26.5319	89.55	80.94	39.70	29.2
TAV8	842,328,290	840,387,539	30,133,268	83,563,752,419	25.0452	80.33	60.65	39.50	28.4

Supplementary Table 3. RNA-seq sequencing statistics.

Samples	Total reads	Mapped reads	Duplicate reads	Mapped bases	Average quality
BAV1	125,507,831	125,507,831	121,878,891	9,541,726,938	34.9
BAV2	193,334,189	193,334,189	187,910,559	14,714,123,086	34.9
BAV3	53,042,982	53,042,982	47,985,074	4,037,469,262	34.2
BAV4	70,672,005	70,672,005	28,732,552	3,084,052,501	35.9
BAV5	68,694,193	68,694,193	25,317,280	2,991,336,057	35.9
BAV6	67,756,730	67,756,730	19,432,036	2,953,930,503	35.9
BAV7	73,687,810	73,687,810	21,166,779	3,211,983,949	36.0
BAV8	96,309,117	96,309,117	35,129,444	4,197,045,649	35.8
TAV1	105,157,436	105,157,436	101,108,810	7,988,604,946	34.5
TAV2	95,752,095	95,752,095	91,278,734	7,271,993,676	34.6
TAV3	71,915,021	71,915,021	31,689,838	3,138,071,053	35.9
TAV4	67,128,661	67,128,661	64,357,308	5,107,055,925	34.2
TAV5	140,855,882	140,855,882	71,036,640	6,126,736,379	36.0
TAV6	84,822,615	84,822,615	39,626,674	3,697,709,351	35.9
TAV7	75,097,792	75,097,792	28,311,296	3,279,824,698	35.9
TAV8	77,036,046	77,036,046	36,554,293	3,360,443,249	35.9

Supplementary Table 4. ChIP-Seq sequencing statistics.

Samples	Total reads	Mapped reads	Duplicate reads	Mapped bases	Average quality
BAVacetyl_1	59,312,503	58,917,898	15,886,389	4,624,690,929	33.4
BAVcontr_1	45,155,915	44,928,269	21,000,087	3,532,736,227	34.1
BAVacetyl_2	76,193,507	75,706,433	20,736,419	5,948,151,184	33.5
BAVcontr_2	57,471,679	56,432,880	36,526,412	4,434,382,266	33.9
TAVacetyl_1	65,271,355	64,659,518	10,122,856	5,081,529,162	33.5
TAVcontr_1	52,445,793	51,376,230	14,421,473	4,037,160,978	33.9
TAVacetyl_2	62,318,425	61,941,142	11,329,901	4,872,268,751	33.8
TAVcontr_2	45,809,560	45,468,290	7,854,867	3,576,961,086	34.0
