# Supplementary material for: Rare regulatory mutations disrupt mesenchymal molecular programs driving endocardial cushion formation in bicuspid aortic valve
Source: Nat Commun. 2026 Apr 18;17:3587. doi: 10.1038/s41467-026-71758-5 (PMC13090386; doi:10.1038/s41467-026-71758-5)
Supplement: Supplementary file 2 — Description of Additional Supplementary Files [file 41467_2026_71758_MOESM2_ESM.pdf]

Description of Additional Supplementary Files

Supplementary Data

File Name: Supplementary Data 1
Description: includes tier 1 and tier 2 gene sets, housekeeping and endothelial-specific genes, as well as BAV- and TAV- specific genes resulting from the analyses.

File Name: Supplementary Data 2
Description: statistically significant HiCap interactions.

File Name: Supplementary Data 3
Description: H3K27ac ChIP-Seq peaks for AECs.

File Name: Supplementary Data 4
Description: allele-specific analysis results.

File Name: Supplementary Data 5
Description: variant annotations with TFs.

File Name: Supplementary Data 6
Description: enformer predictions.

File Name: Supplementary Data 7
Description: probes used for HiCap.

File Name: Supplementary Data 8
Description: HiCap dataset statistics.

File Name: Supplementary Data 9
Description: bulk RNA-seq TPM.
