# Supplementary material for: Rare regulatory mutations disrupt mesenchymal molecular programs driving endocardial cushion formation in bicuspid aortic valve
Source: Nat Commun. 2026 Apr 18;17:3587. doi: 10.1038/s41467-026-71758-5 (PMC13090386; doi:10.1038/s41467-026-71758-5)
Supplement: Supplementary file 12 — Reporting Summary [file 41467_2026_71758_MOESM12_ESM.pdf]

nature portfolio

Corresponding author(s): Pelin Sahlén

Last updated by author(s): 19/02/2025

Reporting Summary

Nature Portfolio wishes to improve the reproducibility of the work that we publish. This form provides structure for consistency and transparency in reporting. For further information on Nature Portfolio policies, see our Editorial Policies and the Editorial Policy Checklist.

Statistics

For all statistical analyses, confirm that the following items are present in the figure legend, table legend, main text, or Methods section.

n/a	Confirmed	
☐	☒	The exact sample size (*n*) for each experimental group/condition, given as a discrete number and unit of measurement
☐	☒	A statement on whether measurements were taken from distinct samples or whether the same sample was measured repeatedly
☐	☒	The statistical test(s) used AND whether they are one- or two-sided<br>*Only common tests should be described solely by name; describe more complex techniques in the Methods section.*
☐	☒	A description of all covariates tested
☐	☒	A description of any assumptions or corrections, such as tests of normality and adjustment for multiple comparisons
☐	☒	A full description of the statistical parameters including central tendency (e.g. means) or other basic estimates (e.g. regression coefficient) AND variation (e.g. standard deviation) or associated estimates of uncertainty (e.g. confidence intervals)
☐	☒	For null hypothesis testing, the test statistic (e.g. *F*, *t*, *r*) with confidence intervals, effect sizes, degrees of freedom and *P* value noted<br>*Give P values as exact values whenever suitable.*
☒	☐	For Bayesian analysis, information on the choice of priors and Markov chain Monte Carlo settings
☐	☒	For hierarchical and complex designs, identification of the appropriate level for tests and full reporting of outcomes
☒	☐	Estimates of effect sizes (e.g. Cohen's *d*, Pearson's *r*), indicating how they were calculated

Our web collection on statistics for biologists contains articles on many of the points above.

Software and code

Policy information about availability of computer code

Data collection	No special software or code was used to collect the data.
Data analysis	Short SNPs and indels calling and annotation: nf-core/sarek/2.7.1 (BWA, GATK HaplotypeCaller) + GATK Variant Quality Score Recalibration algorithm, and annotated with snpEff/4.3t, SweGen/20190204, GnomAD v4.<br>Structural Variants calling and annotation: nf-core/sarek/3.1.2 (Manta), annotated with ensemblVEP/106.1 and snpEff/5.1d.<br>HiCap: bwa-mem2 (version 2.2.1-20211213-edc703f), PairTools (version 1.0.2), HiCapTools.<br>RNA-seq: nf-core/rnaseq/3.5, edgeR.<br>ChIP-seq: nf-core/chipseq/1.2.2.<br>Allele-specific analysis: bcftools (v.1.21), MIXALIME (v.2.25.2).<br>Motif annotation: PERFECTOS-APE v3.0.6.<br>Networks: Cytoscape v3.10.3.<br>Enformer: PyTorch implementation and the HF Accelerate framework.

For manuscripts utilizing custom algorithms or software that are central to the research but not yet described in published literature, software must be made available to editors and reviewers. We strongly encourage code deposition in a community repository (e.g. GitHub). See the Nature Portfolio guidelines for submitting code & software for further information.

Data

Policy information about availability of data

All manuscripts must include a data availability statement. This statement should provide the following information, where applicable:

- Accession codes, unique identifiers, or web links for publicly available datasets
- A description of any restrictions on data availability
- For clinical datasets or third party data, please ensure that the statement adheres to our policy

Raw DNA and RNA sequencing read files generated in this study are regulated by the Swedish Law on Patient Data (2008:355). They are available under controlled access because they are derived from human biological samples and contain individual-level genomic sequence information that may enable participant re-identification. Participant consent permits data sharing only via controlled-access procedures.
Access may be granted to qualified researchers for research purposes upon reasonable request. Requests should include a brief research proposal, intended data use, and confirmation of appropriate ethical approvals. Requests should be directed to the corresponding author hanna.bjorck@ki.se. Requests will be acknowledged within 5 working days and are typically reviewed within 30 days.
Approved applicants will be required to sign a data use agreement restricting use of the data to the approved project and prohibiting attempts to re-identify participants or redistribute the data to third parties.
Intermediate, anonymized data required to replicate the analysis are provided in Supplementary Tables and Supplementary Data. Source data are provided with this paper.

Research involving human participants, their data, or biological material

Policy information about studies with human participants or human data. See also policy information about sex, gender (identity/presentation), and sexual orientation and race, ethnicity and racism.

Reporting on sex and gender	We did not perform analysis stratified based on sex or gender.
Reporting on race, ethnicity, or other socially relevant groupings	We did not perform analyses stratified based on race, ethnicity or other socially relevant groupings.
Population characteristics	15 out of 16 patients with Swedish descent and 1 out 16 patient is of Middle-Eastern descent.
Recruitment	BAV and TAV individuals were undergoing elective open-heart surgery for ascending aortic dilatation at the Cardiothoracic Surgery Unit, Karolinska University Hospital, Stockholm, Sweden.
Ethics oversight	The study was approved by the Human Research Ethics Committee at Karolinska Institutet (application number 2006/784-31/1 and 2012/1633-31/4), Stockholm, Sweden; written informed consent was obtained from all the individuals according to the Declaration of Helsinki, and methods were carried out following relevant guidelines.

Note that full information on the approval of the study protocol must also be provided in the manuscript.

Field-specific reporting

Please select the one below that is the best fit for your research. If you are not sure, read the appropriate sections before making your selection.

☒ Life sciences ☐ Behavioural & social sciences ☐ Ecological, evolutionary & environmental sciences

For a reference copy of the document with all sections, see nature.com/documents/nr-reporting-summary-flat.pdf

Life sciences study design

All studies must disclose on these points even when the disclosure is negative.

Sample size	No statistical methods were used to predetermine sample size. 8 patients with BAV and 8 patients with TAV were included in this study.
Data exclusions	No data were excluded from the analyses.
Replication	All attempts at replication were successful.
Randomization	Randomization was not relevant. This is an observational study, rather than a randomized controlled trial, so no randomization was performed.
Blinding	Blinding was not relevant. This study did not involve subjective evaluations.

Reporting for specific materials, systems and methods

We require information from authors about some types of materials, experimental systems and methods used in many studies. Here, indicate whether each material, system or method listed is relevant to your study. If you are not sure if a list item applies to your research, read the appropriate section before selecting a response.

Materials & experimental systems

n/a	Involved in the study
☒	☐ Antibodies
☒	☐ Eukaryotic cell lines
☒	☐ Palaeontology and archaeology
☒	☐ Animals and other organisms
☒	☐ Clinical data
☒	☐ Dual use research of concern
☒	☐ Plants

Methods

n/a	Involved in the study
☐	☒ ChIP-seq
☒	☐ Flow cytometry
☒	☐ MRI-based neuroimaging

Plants

Seed stocks	NA
Novel plant genotypes	NA
Authentication	NA

ChIP-seq

Data deposition

☐ Confirm that both raw and final processed data have been deposited in a public database such as GEO.

☒ Confirm that you have deposited or provided access to graph files (e.g. BED files) for the called peaks.

Data access links<br>*May remain private before publication.*	Raw DNA and RNA sequencing read files generated in this study are regulated by the Swedish Law on Patient Data (2008:355). Access may be granted to qualified researchers for research purposes upon reasonable request as described in the Data Availability.
Files in database submission	Summary sequencing files, and intermediate, anonymized data required to replicate the analysis are provided in Table S4 and Supplementary Data 3.
Genome browser session<br>(e.g. UCSC)	N/A

Methodology

Replicates	two technical replicates per individual
Sequencing depth	57428833±10322488 single-end reads, 568510±319681 unmapped reads, 17234801±9116459 duplicated reads
Antibodies	H3K27Ac (Diagenode, C15410196-10)
Peak calling parameters	nextflow run /chipseq/1.2.2/workflow --input input.txt --single_end --genome GRCh38 --skip_spp
Data quality	broad peak qvalue cutoff 0.1
Software	nf-core/chipseq v1.2.2
